# One‐year persistence of neutralizing anti‐SARS‐CoV‐2 antibodies in dialysis patients recovered from COVID‐19

**DOI:** 10.1111/hdi.12963

**Published:** 2021-07-06

**Authors:** Gaetano Alfano, Francesco Fontana, Niccolò Morisi, Francesco Giaroni, Giacomo Mori, Giovanni Guaraldi, Riccardo Magistroni, Gianni Cappelli, Modena Covid‐19 Working Group (MoCo19), Cristina Mussini, Giovanni Guaraldi, Erica Bacca, Andrea Bedini, Vanni Borghi, Giulia Burastero, Federica Carli, Giacomo Ciusa, Luca Corradi, Gianluca Cuomo, Margherita Di Gaetano, Giovanni Dolci, Matteo Faltoni, Riccardo Fantini, Giacomo Franceschi, Erica Franceschini, Vittorio Iadisernia, Damiano Larné, Marianna Menozzi, Marianna Meschiari, Jovana Milic, Gabriella Orlando, Francesco Pellegrino, Alessandro Raimondi, Carlotta Rogati, Antonella Santoro, Roberto Tonelli, Marco Tutone, Sara Volpi, Dina Yaacoub, Gianni Cappelli, Riccardo Magistroni, Gaetano Alfano, Francesco Fontana, Giacomo Mori, Elisabetta Ascione, Francesca Damiano, Silvia Giovanella, Giulia Ligabue, Martina Montani, Niccolò Morisi, Andrea Melluso, Monica Frisina, Camilla Ferri, Annachiara Ferrari, Massimo Girardis, Alberto Andreotti, Emanuela Biagioni, Filippo Bondi, Stefano Busani, Giovanni Chierego, Marzia Scotti, Lucia Serio, Andrea Cossarizza, Caterina Bellinazzi, Rebecca Borella, Sara De Biasi, Anna De Gaetano, Lucia Fidanza, Lara Gibellini, Anna Iannone, Domenico Lo Tartaro, Marco Mattioli, Milena Nasi, Annamaria Paolini, Marcello Pinti

**Affiliations:** ^1^ Surgical, Medical and Dental Department of Morphological Sciences, Section of Nephrology University of Modena and Reggio Emilia Modena Italy; ^2^ Nephrology Dialysis and Transplant Unit University Hospital of Modena Modena Italy; ^3^ Clinical and Experimental Medicine PhD Program University of Modena and Reggio Emilia Modena Italy; ^4^ Clinic of Infectious Diseases University Hospital of Modena Modena Italy

**Keywords:** antibody, COVID‐19, dialysis, immunity, Sars‐CoV‐2, vaccine

## Abstract

The immunological mechanisms that modulate immune response to SARS‐CoV‐2 infection remain elusive. Little is known on the magnitude and the durability of antibody response against COVID‐19. There is consensus that patients with immune dysfunction, such as dialysis patients, may be unable to mount a robust and durable humoral immunity after infections. Recent studies showed that dialysis patients seroconverted after COVID‐19, but data on the durability of the immune response are missing. We reported the data of a durable anti‐spike protein seroconversion after natural SARS‐CoV‐2 infection in three patients on hemodialysis with a mean age of 67.2 ± 13.8 years. A mean antibody titer of 212.6 ± 174.9 UA/ml (Liaison®, DiaSorin) was found after one year (range, 366–374 days) from the diagnosis of COVID‐19. In conclusion, this case series provided evidence that patients receiving hemodialysis who recovered from severe COVID‐19 were able to mount a long‐lasting immune response against SARS‐CoV‐2. Although the protective capacity of this long‐term immunity remains to be determined, these patients did not report signs of reinfection after recovery from COVID‐19.

## INTRODUCTION

It is still unclear which immune mechanisms modulate magnitude and duration of the humoral response in patients who contract COVID‐19. This issue became even more challenging in maintenance dialysis patients who are considered as immunocompromised^1^ with lower rates of seroconversion to vaccinations.^2^ Recent evidence shows that most of the hemodialysis patients (95%) are able to seroconvert^3^ and mount a robust antibody response^4^ but data on the long‐term persistence of protecting antibodies are missing.^5^ Here, we report a case series of three patients on dialysis with a durable anti‐spike protein seroconversion after natural SARS‐CoV‐2 infection.

## RESULTS

Two patients on hemodialysis and one on peritoneal dialysis contracted COVID‐19 during the first wave in March 2020. The diagnosis of COVID‐19 was confirmed by a qualitative polymerase‐chain‐reaction assay. Mean age was 67.2 ± 13.8 years. All three patients need hospitalization for severe symptoms of COVID‐19 manifesting principally with fever (100%), dyspnea (66.6%) and hypoxemia (66.6%). Lung involvement was detected in all three patients and multifocal areas of consolidation were found in two of them. The average hospitalization lasted 22.6 ± 14.5 days and nobody required intensive care for worsening of the symptoms associated with COVID‐19. One patient on peritoneal dialysis was switched to hemodialysis via a central vein catheter for failure of peritoneal ultrafiltration. After discharge, this group of patients continued to receive appropriate dialysis therapy in a separated room until the detection of two negative nasopharyngeal swabs for SARS‐CoV‐2. On average, SARS‐CoV‐2 RNA shedding was measured for 44.6 ± 26.1 days; in one patient viral genetic material was detected for 74 days from the diagnosis of COVID‐19 and 79 from symptoms onset.^6^ After the clearance of the virus, no cases of COVID‐19 reinfection were documented in these patients. As detailed in Table 1, anti‐spike IgG antibodies (Ab) were measured in the serum of these three survivors after 1 year (range, 366–374 days) from the diagnosis of the SARS‐CoV‐2 infection. Mean Ab titers were 212.6 ± 174.9 UA/ml. Detection of IgG Ab anti‐trimeric spike glycoprotein was assessed by chemiluminescent immunoassay ([CLIA], Liaison®, DiaSorin).

**TABLE 1:** Demographic and clinical characteristics of COVID‐19 patients

	Case #1	Case #2	Case #3
Age (year)	72.7	77.5	51.5
Sex	M	F	M
BMI	26.4	27.3	30.1
Dialysis modality	PD/HDF^*^	HDF	HDF
Dialysis vintage (year)	2.2	11.2	5.7
No. of comorbidities	3	2	5
*Symptoms*			
Fever	Yes	Yes	Yes
Cough	Yes	Yes	No
Dyspnea	Yes	Yes	No
Diarrhea	Yes	No	No
Myalgia	No	No	Yes
Radiological findings of pneumonia	Yes	Yes	Yes
MAP (mmHg)	80	73.3	101.6
PO_2_ (mmHg)	43	63.9	73.5
Nadir PO_2_ (mmHg)	43	60	54.9
Hospitalization	Yes	Yes	Yes
Duration of hospitalization (days)	9	21	38
Days from symptoms to hospitalization	0	3	2
*Therapy*			
Oxygen therapy	Yes	Yes	Yes
Hydroxychloroquine	No	Yes	Yes
Darunavir/cobicistat	No	Yes	No
Tocilizumab	No	No	Yes
Heparin	No	Yes	No
RNA Shedding from COVID‐19 diagnosis (days)	74	24	36
Last Ab titer of IgG anti‐Spike (UA/ml)	376	28	234
Time elapsed from COVID‐19 to Ab detection (days)	374	369	366

Abbreviations: Ab, antibody; BMI, body mass index; HDF, hemodiafiltration; MAP, mean arterial pressure; PD, peritoneal dialysis. * The patient switched from PD to HDF 29 days after COVID‐19 diagnosis.

## DISCUSSION

Dialysis patients are known to be susceptible to many major infections, which impact patient survival.^1^ There is a prevailing belief that this group of patients has an impaired immune response to foreign antigens. A blunted immune response is indeed seen after some vaccination cycle (e.g., influenza, HBV), especially in elderly subjects.^2^ As a result, adapted immunization protocols with larger doses are often required to effectively seroconvert this population.

Because of the aforementioned issues, the nephrology community is uncertain whether hemodialysis patients can maintain a durable seroconversion after SARS‐CoV‐2 infection.^5^ This case series showed that SARS‐CoV‐2 infection provided long‐lasting humoral immunity in three patients undergoing maintenance dialysis. Hence, our data confirm the recent encouraging findings on the protective immunity against COVID‐19, showing that natural infection induces robust memory B‐cell response lasting at least 8 months in the general population.^7^ However, work is still needed to ascertain if antibodies against SARS‐CoV‐2 are able to protect from severe COVID‐19. This is issue is particularly relevant for hemodialysis patients that have a low level of antibodies as in our case #2. Emerging evidence suggests that neutralizing antibodies after SARS‐CoV‐2 infection are associated with protection against reinfection,^8^ notably, the presence of anti‐spike or anti‐nucleocapsid IgG antibodies portends a substantially lower risk of reinfection in the following 6 months from the primary infection.^9^

However, many variable can affect immune response against SARS‐CoV‐2 infection. We suppose that multiple determinants including nonmodifiable (sex, age, ethnicity) and clinical (illness severity, dialysis vintage, comorbidities, immune dysfunction) factors and socioeconomic status play a key role in the kinetics of humoral response in dialysis patients and may determine the basis of a heterogeneous antibody production. While awaiting data from ongoing observational studies, durable humoral immunity should be considered a good sign for vaccination policy^10^ and the future course of the COVID‐19 pandemic in this group of vulnerable patients.

## CONFLICT OF INTEREST

All the authors declared no competing interest.

## ETHICS STATEMENT

This study has been authorized by the local Ethical Committee of Emilia Romagna (protocol number: AOU 0010159/20‐0021170/20). The study was conducted in accordance with the Helsinki Declaration.
